# Preclinical Evaluation of Radiolabeled Peptides for PET Imaging of Glioblastoma Multiforme

**DOI:** 10.3390/molecules24132496

**Published:** 2019-07-08

**Authors:** Zbynek Novy, Jana Stepankova, Michaela Hola, Dominika Flasarova, Miroslav Popper, Milos Petrik

**Affiliations:** Institute of Molecular and Translational Medicine, Faculty of Medicine and Dentistry, Palacky University Olomouc, 77900 Olomouc, Czech Republic

**Keywords:** PET, RGD peptides, biodistribution, gallium-68, ^18^F-FDG, ^18^F-FLT, glioblastoma multiforme

## Abstract

In this study, we have compared four ^68^Ga-labeled peptides (three Arg-Gly-Asp (RGD) peptides and substance-P) with two ^18^F-tracers clinically approved for tumor imaging. We have studied in vitro and in vivo characteristics of selected radiolabeled tracers in a glioblastoma multiforme tumor model. The in vitro part of the study was mainly focused on the evaluation of radiotracers stability under various conditions. We have also determined in vivo stability of studied ^68^Ga-radiotracers by analysis of murine urine collected at various time points after injection. The in vivo behavior of tested ^68^Ga-peptides was evaluated through ex vivo biodistribution studies and PET/CT imaging. The obtained data were compared with clinically used ^18^F-tracers. ^68^Ga-RGD peptides showed better imaging properties compared to ^18^F-tracers, i.e., higher tumor/background ratios and no accumulation in non-target organs except for excretory organs.

## 1. Introduction

Glioblastoma multiforme (GBM) is an aggressive primary brain tumor and, despite the current intensive therapy regimen, median overall survival period is only 16–21 months from the date of diagnosis [1]. Therefore, early and precise diagnosis of GBM is a key aspect for appropriate patient care approach [2]. Imaging techniques play an important role in the diagnosis of GBM and subsequent disease monitoring. Although magnetic resonance imaging (MRI) is the first-line technique for GBM imagining, positron emission tomography (PET) is increasingly used in gliomas diagnosis as well [3,4]. The diagnostic information acquired with ^18^F-fluorodeoxyglucose (^18^F-FDG), the gold standard in clinical PET tumor imaging, is relatively variable; however, amino acid PET tracers, such as ^11^C-methionine (^11^C-MET), ^18^F-fluoro-L-DOPA (^18^F-FDOPA) or ^18^F-fluoroethyltyrosine (^18^F-FET) allow a more accurate glioma characterization [5]. The better tumor characterization could also be the case of more recent radiotracers developed for PET imaging of α_v_β_3_ integrins [4,6]. α_v_β_3_ integrin is highly expressed in glioma cells and generally in neovascular endothelial cells, in conjunction with tumor-related angiogenic processes [7]. The expression rate of α_v_β_3_ integrin increases with the malignancy grade of the glioma and is particularly important in high-grade gliomas, where this integrin may facilitate tumor progression [8]. The radiotracers that bind α_v_β_3_ integrin contain one or more tripeptide Arg-Gly-Asp (RGD) amino acid sequences providing high affinity towards α_v_β_3_ integrins [9,10,11,12]. Although some of these RGD peptides were labeled with fluorine-18, this approach appears not to be convenient for practical use in terms of demanding radiochemical synthesis despite its great diagnostic potential [10,13].

The preparation of RGD peptides, labeled with radiometals like gallium-68 via a metal complexing approach, is much more straightforward in preclinical as well as in clinical settings [6,12]. Recently, RGD peptide coupled with a suitable macrocyclic chelator (e.g., dimeric ^68^Ga-NOTA-PRGD_2_ peptide) was successfully tested in human gliomas and showed better properties than ^18^F-FDG in differentiating high and low-grade gliomas [6]. Apart from RGD peptides and ^18^F-FDG, radiolabeled substance P that targets NK-1 receptors is another promising approach for imaging GMB [14]. In addition to the aforementioned tracers, there were also attempts to visualize glioblastoma with ^18^F-FLT [15].

Nevertheless, a comparison of ^18^F-FDG has never been performed to date with NODAGA-c(RGDyK)_2_, a dimeric RGD peptide employing NODAGA as the chelating moiety, with RGD monomer DOTA-c(RGDfK) and DOTA-substance P in preclinical GBM imaging. Despite the fact that the affinity for α_v_β_3_ integrins is lower for monomeric and dimeric than multimeric RGD peptides, monomeric and dimeric RGD peptides usually have more favorable pharmacokinetic and imaging properties in vivo. The smaller size of mono- and dimeric RGD peptides result in faster pharmacokinetics and are often a more suitable biodistribution profile for tumor imaging [11,14]. In this study, we have focused on the preclinical evaluation of ^68^Ga-labeled peptides (DOTA-c(RGDfK), NODAGA-c(RGDyK), NODAGA-c(RGDyK)_2_, and DOTA-substance P) and comparison of their in vivo behavior with two clinically established ^18^F-based tumor radiotracers (^18^F-FDG and ^18^F-FLT) in a mouse xenograft model of human glioblastoma. Our glioblastoma model is based on U-87 MG cell line, a well-established tumor model expressing α_v_β_3_ integrins and NK-1 receptors [16,17].

## 2. Results

### 2.1. Radiolabeling of Studied Peptides

The radiochemical purity of ^68^Ga-labeled peptides determined by HPLC was 95%. The lowest radiochemical purity of 96.3% was reported for ^68^Ga-DOTA-substance P, while the highest radiochemical purity of 99.8% was observed for ^68^Ga-DOTA-c(RGDfK)

### 2.2. In Vitro Characterization

The results of in vitro evaluation of ^68^Ga-labeled peptides are presented in Table 1. All four studied peptides appeared as hydrophilic entities having partition coefficient values around -3. The three studied RGD peptides showed low plasma protein binding values ranging from 1% to 3%, while substance P displayed slightly different results with approximately 29% fraction bound to the plasma proteins. Another tested in vitro parameter was the stability of ^68^Ga-peptides in the presence of an excess of Fe^3+^ (0.1 M FeCl_3_). RGD peptides showed high stability of 95–99% under these conditions. However, the stability of ^68^Ga-DOTA-substance-P in the presence of a competing metal was 79% after 120 min of incubation. In the presence of an excess concentration of another competing chelator (6 mM DTPA), RGD peptides displayed stability of 99%, while the stability of substance P decreased to 56% after 120 min of incubation. The results of stability test in neutral pH and human plasma were in the range of 98–99% for all four tested ^68^Ga-peptides (Table 1).

### 2.3. In Vitro Uptake

**Figure 1:**
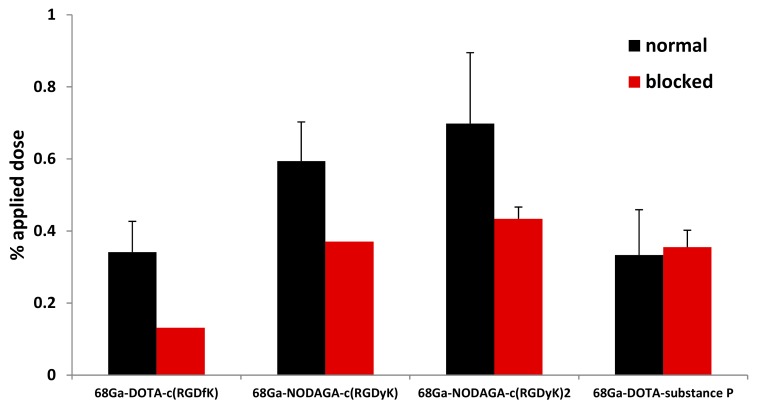
Accumulation of labeled peptides in normal U-87 MG cells (black bars) and U-87 MG cells blocked with an excess of appropriate “cold” RGD peptide (red bars). Data are presented as the mean of the percentage of applied dose ± standard deviation (n = 4).

Uptake studies using U-87 MG cell line showed the accumulation of approximately 0.4–0.8% of the applied dose in cells, with the highest accumulation observed for ^68^Ga-NODAGA-c(RGDyK)_2_ and the lowest for ^68^Ga-DOTA-c(RGDfK) (Figure 1). The presence of an excess of a cold ligand resulted in the decrease of the uptake of radiolabeled ligand in all tested ^68^Ga-RGD peptides except ^68^Ga-DOTA-substance P, which had an uptake of 0.3% of the applied dose under both normal and blocked variants of the experiment. For details, see Figure 1.

### 2.4. In Vivo Stability of ^68^Ga-Peptides

**Figure 2:**
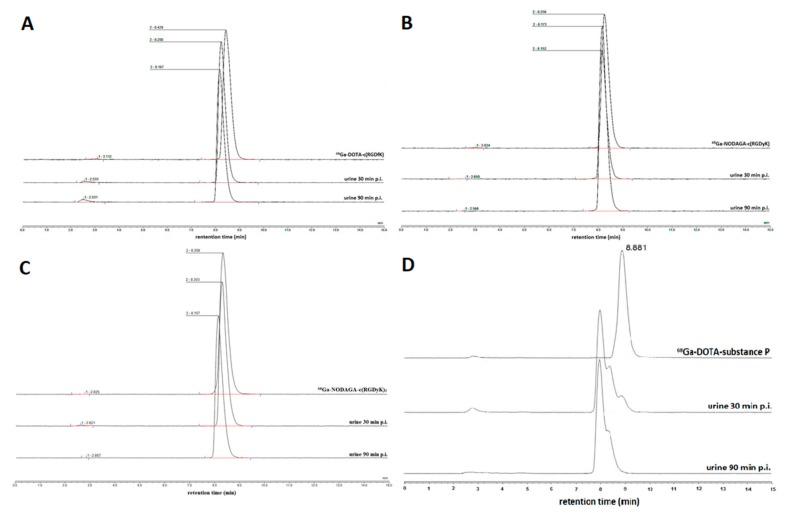
HPLC radiochromatograms of (**A**) ^68^Ga-DOTA-c(RGDfK), (**B**) ^68^Ga-NODAGA-c(RGDyK), (**C**) ^68^Ga-NODAGA-c(RGDyK)_2_, and (**D**) ^68^Ga-DOTA-substance P. The upper line in every chromatogram represents the tracer before the application to animals; the other two are samples of urine 30 min p.i. and 90 min p.i.

The urine of animals injected with ^68^Ga-peptides was collected 30 and 90 min after the administration for HPLC analysis, and the respective radiochromatograms of ^68^Ga-peptides and urine are summarized in Figure 2. The HPLC analysis of ^68^Ga-RGD peptides compared to appropriate urine samples showed no major shift in the retention time and the shape of the chromatographic peaks is almost similar to the peaks of observed with original peptides and urine samples. The percentage of radiochemical impurities is practically negligible. However, the urine samples of animals injected with ^68^Ga-DOTA-substance P show a clear shift in the retention time of main peak (approximately 1 min) and appearance of new peaks suggesting the formation of more hydrophilic metabolites.

### 2.5. Ex Vivo Biodistribution

**Figure 3:**
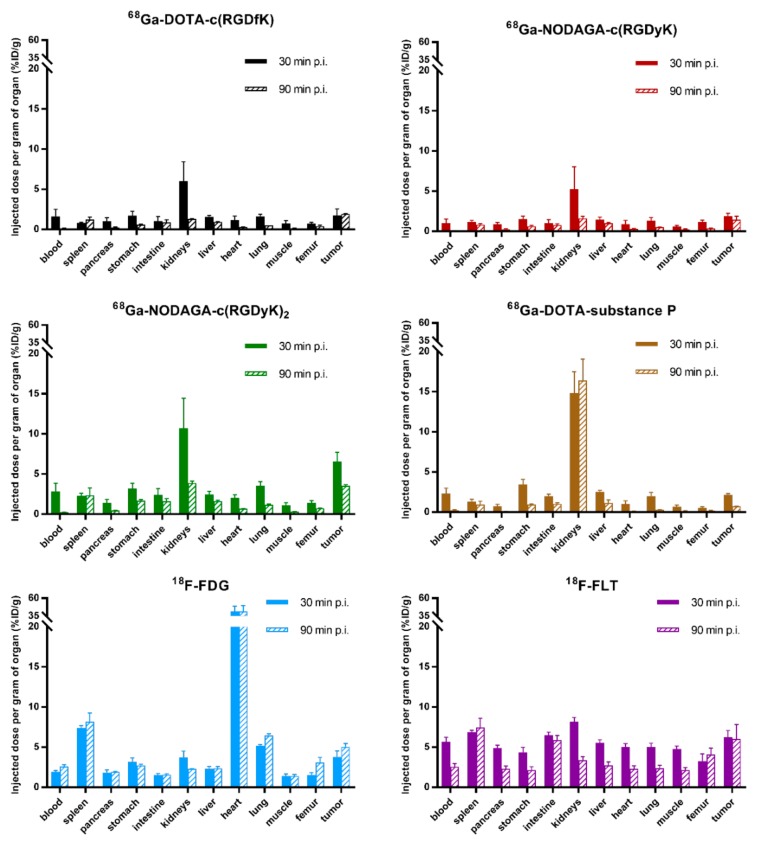
Ex vivo biodistribution of ^68^Ga-peptides and ^18^F-tracers in U-87 MG tumor SCID mouse 30 and 90 min p.i. The data are presented as the mean of the percentage of injected dose per gram of organ ± standard deviation (n = 3).

The biodistribution results were similar for all four tested ^68^Ga-peptides showing very low accumulation of the tracers in non-targeted tissues (Figure 3). The highest radioactive accumulation was registered in kidneys and ranged between 6–11% ID/g for ^68^Ga-RGD peptides and 15–17% for ^68^Ga-DOTA-substance P. In summary, all tested ^68^Ga-labeled peptides showed rapid pharmacokinetics with the main renal excretion route and very low accumulation in non-targeted tissues. Ex vivo biodistribution data for ^18^F-FDG displayed relatively low accumulation in non-targeted tissues (1–8% ID/g) with the exception of heart, where the accumulation reached 40% ID/g. ^18^F-fluorothymidine (^18^F-FLT) showed uniform accumulation and retention of 5–8% ID/g in all examined tissues for 30 and 90 min post injection (p.i.). Both ^18^F-tracers had relatively higher accumulation in non-targeted tissues and slower elimination from blood compared to ^68^Ga-compounds.

Comparative tumor to blood/kidney/muscle ratios were calculated from the data obtained from ex vivo biodistribution studies (Table 2). Tumor to blood ratios 30 min p.i. were in the range of 1–2.3 for all tested ^68^Ga-peptides. Ninety minutes p.i., the tumor to blood ratio values ranged from 14–23 for ^68^Ga-RGD peptides and 2.8 for ^68^Ga-substance P. Similarly, the tumor to blood ratio 30 min and 90 min p.i. for ^18^F-FDG was 2.0 for both time points and 1.1 and 2.4, respectively, for ^18^F-FLT.

### 2.6. µPET/CT Imaging

**Figure 4:**
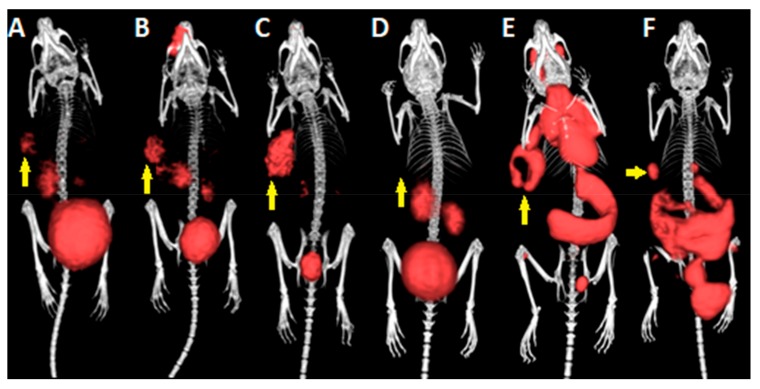
3D volume rendered PET/CT images of (**A**) ^68^Ga-DOTA-c(RGDfK), (**B**) ^68^Ga-NODAGA-c(RGDyK), (**C**) ^68^Ga-NODAGA-c(RGDyK)_2_, (**D**) ^68^Ga-DOTA-substance P, (**E**) ^18^F-FDG and (**F**) ^18^F-FLT in mice bearing U-87 MG xenograft tumor on the right flank 90 min p.i. of the tracer. Yellow arrows are indicating the position of the tumor.

PET/CT imaging of ^68^Ga-labeled peptides clearly showed the highest accumulation of tracer in the kidneys and bladder U-87 MG tumor xenografts were visualized with all three ^68^Ga-RGD peptides, with the highest accumulation of radioactivity noted for ^68^Ga-NODAGA-c(RGDyK)_2_. ^68^Ga-substance P did not visualize the tumor in any of the studied time intervals. PET/CT of U-87 MG tumor xenografts with ^18^F-FDG tracer allowed clear imaging of the tumor; however, high radioactivity signal was also observed in non-target tissues such as spleen, heart, brain, and brown adipose tissue. ^18^F-FLT PET/CT imaging of U-87 MG xenografted mice 90 min p.i. showed the accumulation of the tracer mainly in the gastrointestinal tract, spleen, and bladder. The tumor on the right flank of the animal could also be imaged with ^18^F-FLT. The acquired three-dimensional PET/CT images are shown in Figure 4. Using acquired PET data we have determined standard uptake values (SUV) for the tumor and brain regions and calculated tumor-to-brain ratios listed in Table 3. These ratios have ranged from 0.64 up to 4.70.

## 3. Discussion

MRI imaging and tumor biopsy still remain as the diagnostic gold standard for GBM. Nevertheless, various studies now confirm the diagnostic potential and advantages of PET imaging for GBM patients due to better tumor delineation or monitoring tumor progression [18,19]. Recently, radiolabeled RGD peptides were successfully used to image other human carcinomas with PET technology [20] and RGD-sequence peptides remain emerging candidates for glioblastoma imaging [21,22,23].

In the past 20 years, RGD peptides underwent relatively extensive preclinical development, beginning with ^18^F-based ^18^F-galacto-RGD peptide [12]. This promising tracer was substituted with ^68^Ga-labeled variants due to its better targeting properties and more simple synthesis. The cyclization of the structure and its multimerization was also part of the development, which indicated that dimerization of the structure and cyclic form show the best in vivo imaging results. The multimeric (four and more moieties) RGD structure has the disadvantage of slower pharmacokinetics and higher image background. Among the most studied RGD-based tracers, RGDyK and RGDfK sequences, the published data are more favorable towards RGyK due to its faster renal elimination [24]. To our knowledge, there are very few complex reports focusing on the comparison of traditional ^18^F-tracers with ^68^Ga-labeled RGD peptides for glioblastoma imaging. In this comparative study, we reveal the potential of ^68^Ga-RGD peptides under preclinical settings and show their advantages over clinically used ^18^F-FDG and ^18^F-FLT. We cover here the whole issue ranging from radiolabeling, in vitro characterization, in vivo biodistribution studies to PET/CT imaging of U-87 MG xenografted tumors in mice.

The radiolabeling of studied peptide tracers with ^68^Ga resulted in excellent radiochemical purity (97–99%) for all RGD peptides and slightly lower value for ^68^Ga-substance P (96%). DOTA modified tracers required elevated reaction temperatures (95 °C), while NODAGA-modified tracers provided excellent labeling results at room temperature. Log P values of all peptide tracers indicated that these molecules are highly hydrophilic, which was subsequently confirmed by animal experiments.

The plasma protein binding values of all three tested RGD peptides were very low (1–3%), except ^68^Ga-DOTA-substance P that showed a higher (about 29%) protein binding value, what is in accordance with previously published data [12]. Nevertheless, we did not register much higher blood levels of ^68^Ga-DOTA-substance P in animals compared to ^68^Ga-RGD peptides. In the presence of an excess of competing chelator (DTPA), all tested ^68^Ga-RGD peptides displayed excellent stability (99.06–99.65%), while ^68^Ga-DOTA-substance P had an average stability. The presence of an excessive amount of Fe^3+^ ions had lower effect on radiochemical purity, resulting in the lowest stability again for ^68^Ga-DOTA-substance P. These findings confirmed previously published data, indicating that NODAGA has better chelating properties for ^68^Ga^3+^ compared to DOTA [12]. Additionally, all ^68^Ga-peptides showed excellent stability under physiological pH and in human plasma, with a negligible decrease in the radiochemical purity. These findings are in general in accordance with previous published data [12].

The specificity of tracer binding to U-87 cells was tested by incubating the cells with an excess concentration of a cold tracer. The highest binding was detected for ^68^Ga-NODAGA-c(RGDyK)_2_, while ^68^Ga-DOTA-c(RGDfk) and ^68^Ga-DOTA-substance P showed the lowest uptake of about 50% compared to ^68^Ga-NODAGA-c(RGDyK)_2_. This is in accordance with previous studies that showed the benefits of multimerization and the superiority of RGDyK sequence over RGDfK [11,25]. The RGD dimer possesses higher cell binding property compared to monomer as shown by Li et al. [11]. Blocking the in vitro uptake of ^68^Ga-tracers by cold ligand was successful for all RGD peptides except ^68^Ga-DOTA-substance P, despite the fact that U-87 MG cells express neurokinin (NK1) receptor, the target of substance P [26].

Determination of in vivo stability of studied tracers in mice urine showed that RGD peptides are excreted in unchanged form, while ^68^Ga-DOTA-substance P is rapidly metabolized and excreted in different forms compared to the parent molecule. This is clearly visible in radiochromatograms (Figure 2), where the ^68^Ga-substance P peak is shifted and branched in urine samples. HPLC analysis of urine from ^68^Ga-RGD peptides-injected animals showed a similar radiochromatogram as radiolabeled peptide. These findings indicate that ^68^Ga-DOTA-substance P have a poor in vivo stability compared to other tested ^68^Ga-RGD peptides.

Ex vivo biodistribution studies revealed the highest uptake of ^68^Ga-tracers in kidneys as the principal organ of their excretion, where ^68^Ga-substance P had the highest uptake of 15% ID/g. The GBM tumors had relatively low uptake values of about 2% ID/g, except for ^68^Ga-NODAGA-c(RGDyK)_2_ with 6% ID/g for 30 min p.i. These findings are in accordance with previously published data by other research groups, where tumor accumulation for RGD peptides ranges from 2.5% to 4% ID/g [11,27]. Ex vivo biodistribution data for ^68^Ga-DOTA-substance P showed relatively low uptake across all studied organs with exception of kidneys, the tumor uptake 30 min p.i. was 2.14% ID/g; compared with the work of Mozaffari et al., we obtained very similar results. However, the tumor uptake in the article was 3.36% ID/g, i.e., higher when compared to our data, in what could be explained by the structural difference of studied compounds [28]. The accumulation of RGD peptides in tumors appears to be quite a fast process. This is indicated by the maximum tumor uptake of ^68^Ga-NODAGA-c(RGDyK)_2_ in 30 min p.i. Tumor to blood and tumor to muscle ratios are the best ex vivo parameters for prediction of an appropriate tumor to background contrast, which can result in suitable images. In this respect, ^68^Ga-NODAGA-c(RGDyK) showed the best tumor to blood ratio of 23.1 in 90 min p.i. All three tested ^68^Ga-RGD peptides revealed excellent tumor to blood ratios (14.7–23.1), in contrast to ^68^Ga-DOTA-substance P (2.8). The best results for tumor to muscle (background) ratio was observed for ^68^Ga-DOTA-c(RGDfK). ^18^F-FDG biodistribution displayed typical high uptake of the tracer in the heart (almost 42% ID/g) and also in the spleen, what is in line with published data [29]. Finally, ^18^F-FLT exhibited relatively uniform accumulation across all studied organs (4–6% ID/g). Nevertheless, this was expected according to the literature [30]. The decrease of tracer uptake between 30 and 90 min post injection was apparent, but it was not the case for spleen, intestine, bone and tumor, On the contrary to ^68^Ga-tracers, ^18^F-tracers had tumor to blood ratio of 2.0. Tumor to kidney ratio was low for all studied ^68^Ga-labeled compounds and all time points, where the lowest values were for ^68^Ga-DOTA-substance P. ^18^F-tracers had lower tumor to muscle ratios and higher tumor to kidney ratio in comparison to ^68^Ga-tracers. These results are fully in accordance with the main excretion route of all ^68^Ga-peptides.

In accordance with our ex vivo data, PET/CT imaging with ^68^Ga-tracers showed kidneys and bladder as the organs with the highest accumulation of radioactivity. However, the tumors were clearly visualized using the three studied ^68^Ga-RGD peptides. The best tumor images were obtained with ^68^Ga-NODAGA-c(RGDyK)_2_, whereas the other two ^68^Ga-RGD peptides displayed lower radioactivity signal in the tumor. These results are in concordance with PET RGD imaging data published by Li et al. and Provost et al. [11,23]. We did not observe any substantial signal in tumors with ^68^Ga-substance P. ^18^F-FDG PET scan was also in the line with our ex vivo data and previously published imaging data, showing predominant accumulation of radioactivity in the heart, spleen, tumor and interscapular brown adipose tissue [23]. Similarly, data from ^18^F-FLT PET imaging were in congruence with ex vivo results with the highest activity seen in the spleen, gastrointestinal tract, tumor, and largest bone tissue sites, such as knees, what confirms the findings published by Chan et al. [30]. The data gained from PET acquisitions were also used for the uptake quantification of the tracers in tumor and brain regions by calculation of standardized uptake values (SUV). Using the SUVs, we have calculated tumor to brain ratios in order to reveal potential tumor to background ratio for the situation of orthotopic glioblastoma. Interestingly, all the ratios ranged between 4 and 5 with the exception of ^18^F-FDG (0.64). These results suggest that ^18^F-FDG is not an appropriate tracer for intracranial tumor imaging because of the high background of the tissue. Surprisingly, ^68^Ga-DOTA-substance P had comparable tumor to brain ratio as ^68^Ga-RGD peptides and ^18^F-FLT; however, the SUVs were very low in both tumor and brain regions in the case of ^68^Ga-substance P. Among ^68^Ga-RGD peptides and ^18^F-FLT, all the tumor to brain ratios were very similar not preferencing any of the studied tracers. Nevertheless, the highest value (4.70) of tumor to brain ratio was registered for ^68^Ga-DOTA-c(RGDfK). In summary, ^68^Ga-RGD peptides showed much favorable kinetics and biodistribution compared to ^18^F-FDG and ^18^F-FLT. Despite relatively low tumor to blood ratio, tumors could be imaged on PET scans with ^18^F-tracers presumably due to the high tumor uptake.

The accumulation of radiolabeled RGD peptides as ligands of α_v_β_3_ integrins reflect the expression rate of these receptors and the tracer could monitor tumor progression and its metastases [31]. The integrin receptors are saturable as was shown in our in vitro cell study. Therefore, it is important to inject the lowest possible dose (low molar amount of RGD peptide) to avoid saturation of target receptors with the cold fraction of the peptide and consecutive decrease in the detected signal in tumor tissues. In contrast to ^68^Ga-RGD peptides, ^18^F-tracers do not image the rate of integrin expression but only the metabolic rate of compounds (glucose or thymidine). The metabolic rate is also a very useful characteristic of a tumor and could be used to predict various biological characteristics [32].

In conclusion, our study confirmed α_v_β_3_ integrins as a good target for GBM imaging using ^68^Ga-RGD peptides that have a favorably biodistribution profiles and a rapid pharmacokinetics. RGD peptides are very versatile and are not exclusive for GBM as they can image various other malignancies, such as breast cancer, rectal cancer, NSCLC. [33]. The possibility of radiolabeling ^68^Ga-labeled RGD peptides in laboratories without the need an on-site cyclotron facility give them an undisputed advantage over ^18^F-tracers.

## 4. Materials and Methods

### 4.1. Chemicals

**Figure 5:**
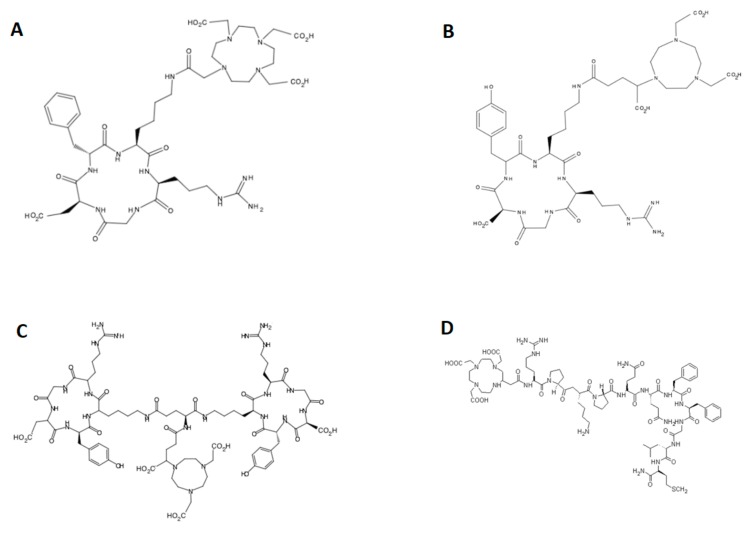
The chemical structures of tested peptides coupled with respective chelators. (**A**) DOTA-c(RGDfK), (**B**) NODAGA-c(RGDyK), (**C**) NODAGA-c(RGDyK)_2_, and (**D**) DOTA-substance P.

All reagents were purchased from commercial sources as analytical grade and used without further purification. Two clinically used radiopharmaceuticals were employed in this study, ^18^F-fluorotymidine (RadioMedic, Rez, Czech Republic) and ^18^F-fluorodeoxyglucose (UJV, Rez, Czech Republic), for comparison with non-RGD peptide DOTA-substance P and RGD peptides coupled with chelators, namely DOTA-c(RGDfK), NODAGA-c(RGDyK) and NODAGA-c(RGDyK)_2_. and. All “cold” peptides were purchased from ABX-Advanced biochemical compounds (Radeberg, Germany) with >95% purity. The structures of these four peptides are shown in Figure 5. ^68^GaCl_3_ was eluted from a ^68^Ge/^68^Ga generator (Eckert & Ziegler Eurotope GmbH, Berlin, Germany) with 0.1 N HCl using a fractionated elution approach.

### 4.2. Radiolabeling and in Vitro Characterization of Studied Peptides

Gallium-68 was obtained from ^68^Ge/^68^Ga generator (Eckert & Ziegler, Berlin, Germany) by elution with 0.1 M HCl (Sigma Aldrich, St. Louis, MO, USA). The optimal ^68^Ga-labeling conditions for all peptides were evaluated as follows: 5 μg of peptide (1 μg/μL) dissolved in LC-MS ultrapure water (Sigma Aldrich, St. Louis, MO, USA) mixed with 30 μL of 1.14 M sodium acetate trihydrate (Merck, Darmstadt, Germany) and with 300 µL of ^68^Ga eluate. The mixture was incubated for 10 min at RT for RGD peptides with NODAGA and at 90 °C for DOTA modified tracer. For substance P, the labeling conditions were slightly different: 10 μg of substance P, temperature of 95 °C, incubation time of 15 min. The radiochemical purity of ^68^Ga-peptides was determined by HPLC analysis employing gradient elution (water to acetonitrile). The HPLC method was described in detail previously [12].

#### 4.2.1. Stability in the Presence of Competing Metal

50 μL of ^68^Ga-labeled peptides were mixed with 150 μL of 0.1 mM FeCl_3_ (Sigma Aldrich, St. Louis, MO, USA). The mixture was incubated at 37 °C and the samples for HPLC analysis to determine the radiochemical purity were taken at selected time points (30, 60 and 120 min) and directly injected on HPLC.

#### 4.2.2. Stability in the Presence of Competing Chelator

50 μL of ^68^Ga-labeled peptides were mixed with 150 μL of 6 mM DTPA solution (Sigma Aldrich, St. Louis, MO, USA). The mixture was then incubated at 37 °C and the samples were processed for HPLC analysis to determine the radiochemical purity were taken at selected time points (30, 60, and 120 min) and directly injected on HPLC.

#### 4.2.3. Stability in Physiological pH

The peptides were labeled as described above. Subsequently, 100 μL of 1.14 M sodium acetate was added to adjust the pH to 7.0. The mixture was incubated at 37 °C and the samples for HPLC analysis to determine the radiochemical purity were taken at selected time points (30, 60, and 120 min) and directly injected on HPLC.

#### 4.2.4. Plasma Stability

100 μL of the labeled peptide was mixed with 400 μL of human plasma (Sigma Aldrich, St. Louis, MO, USA). This mixture was incubated at 37 °C and the samples for HPLC analysis to determine the radiochemical purity were taken at selected time points (30, 60, and 120 min). The individual samples were adjusted before the injection on HPLC—every sample was mixed with acetonitrile (Roth, Karlsruhe, Germany) and placed on the shaker (450 rpm) for 60 s and then centrifuged (20,000 RCF, 3 min), followed by removing the supernatant. Finally, the supernatant was analyzed using HPLC.

#### 4.2.5. Determination of Partition Coefficient (Log P)

Labeled peptide was diluted with 600 μL of phosphate-buffered saline (PBS), and 50 µL of the solution was mixed in the test tube with 450 μL of PBS and 500 μL of octanol. The sample was placed on the shaker (1000 RPM) for 20 min and then centrifuged at 15,000 RPM for 1 min. Subsequently, 50 µL from both octanol and water layer were collected and measured on an automatic gamma counter (PerkinElmer, Waltham, Massachusetts, USA). Partition coefficient was calculated as the decadic logarithm of the ratio of octanol/water activity.

#### 4.2.6. Plasma Protein Binding

10 µL of the labeled peptide was mixed with 190 μL of human plasma (Sigma Aldrich, St. Louis, MO, USA), and incubated at 37 °C. Twenty-five µL of sample were collected at 30, 60, and 120 min time intervals for the determination of protein binding. The samples were separated by size-exclusion chromatography (illustra MicroSpin G-50 Column, GE Healthcare, UK) by centrifugation (2000 × *g*, 1 min). The eluate (protein-bound) and appropriate column (non-protein-bound) were measured on an automatic gamma counter. The percentage of plasma protein binding (PPB) was calculated as the ratio of the activity in the eluate and the activity of the whole sample (column plus the eluate). The blank determination of protein binding, i.e., incubation of the radiotracer in PBS buffer followed by separation of the sample on gel column was performed as the control step.

#### 4.2.7. Determination of Metabolic Stability in Vivo

The experimental animals were intravenously injected with tested radiolabeled peptides (5 MBq corresponding to 1 μg of the peptide per animal) and urine was collected 30 and 90 min post-injection. The urine samples were directly analyzed on HPLC using the same method as for other stability assays. The obtained radiochromatograms were compared with the radiochromatograms of studied ^68^Ga-peptides. The goal of this experiment was to determine whether the peptides are metabolized in vivo or are excreted in unchanged form.

### 4.3. Cell Culture and Cell Uptake Assay

Human glioblastoma multiforme U-87 MG cells (ATCC, Manassas, VA, USA) were cultured in Dulbecco’s Modified Eagle Medium (Sigma Aldrich, St. Louis, MO, USA) supplemented with 10% fetal bovine serum, 0.1 mM non-essential amino acids and 1.0 mM sodium pyruvate at 37 °C in a 5% carbon dioxide humidified incubator. The cells were subcultured and used for experiments at a confluency of 70–90%.

Cell uptake assay was performed in 24-well plates with U-87 MG cell at high confluency. The assay itself was carried out using a dedicated buffer instead of the regular cell culture media. The buffer consists of 25 mM Tris/HCl, 5.4 mM KCl, 1.8 mM CaCl_2_, 0.8 mM MgSO_4_, 5 mM glucose, 140 mM NaCl in H_2_O [34].

The cells were incubated with ^68^Ga-labeled RGD peptides (20 µM) alone and also with excess (2 mM) of cold RGD peptide for 90 min (37 °C) in the buffer described above. The cells were rinsed with PBS, lysed in 0.1 M NaOH and measured for radioactivity in a gamma counter (PerkinElmer, Waltham, Massachusetts, USA). The uptake was calculated as the mean of the percentage of the applied dose ± standard deviation.

### 4.4. Tumor Xenografts

SCID female mice (Harlan, Indianapolis, IN, USA) at 6–8 weeks of age were subcutaneously injected into the right flank with 5 × 10^6^ U-87 MG cells mixed with Matrigel Matrix (Corning, Corning, NY, USA) at a 1:1 ratio. The tumor growth was continuously monitored by caliperation. When the tumor volume reached 100–300 mm^3^ (i.e., 6–8 weeks after inoculation of cells), the mice were used for ex vivo biodistribution studies or µPET/CT imaging. The experimental animals were housed in a specific-pathogen-free animal facility. All experiments with animals were performed in accordance with appropriate legal norms (Czech Law No. 246/1992) and with the approval of Ministry of Education, Youth and Sports (MSMT-18933/2013-1 and MSMT-22421/2013-12) and approval of Ethical committee of Faculty of Medicine and Dentistry, Palacky University in Olomouc. The number of animals was reduced as much as possible (n = 3 per group and time point) for all in vivo experiments. The tracer injection and small animal imaging were carried out under 2% isoflurane anesthesia (FORANE, Abbott Laboratories, Abbott Park, IL, USA) to minimize animal suffering and to prevent animal motion.

### 4.5. Ex Vivo Biodistribution

For ex vivo biodistribution studies, radiolabeled tracers were diluted with saline. The prepared solutions were applied retro-orbitally (r.o.) to the mice [35] at a dose of 1–2 MBq per mouse corresponding to 0.2–0.4 µg of the peptide. The mice were sacrificed by cervical dislocation 30 min and 90 min post-injection and organs of interest (blood, spleen, pancreas, stomach, intestine, kidneys, liver, heart, lungs, muscle, bone, and tumor) were collected. The samples were weighed and the radioactivity was measured by an automatic gamma counter. The accumulation of radiotracer in studied organs was expressed as a percentage of injected dose per gram of organ.

### 4.6. µPET/CT Imaging

Experimental animals were imaged using microPET/SPECT/CT system Albira (Bruker, Billerica, Massachusetts, USA). Mice were r.o. injected with the radiolabeled tracer at a dose of 5–10 MBq (corresponding to 1–2 μg of peptide for animals injected with ^68^Ga-peptides) per animal. Anesthetized animals were placed in a prone position in the Albira system before the start of imaging. Static PET/CT images were acquired over 30 min interval starting 30 and 90 minutes after injection. A 10-min PET scan (axial FOV 148 mm) was performed, followed by a double CT scan (axial FOV 65 mm, 45 kVp, 400 μA, at 400 projections). Scans were reconstructed with the Albira software (Bruker Biospin Corporation, Woodbridge, CT, USA) using the maximum likelihood expectation maximization (MLEM) and filtered back-projection (FBP) algorithms. The PMOD software (PMOD Technologies, Zurich, Switzerland) was used for the image analysis and tracer uptake quantification. Three-dimensional volume rendered images were created in VolView software (Kitware, Clifton Park, NY, USA). The data obtained by PET scan were quantified in PMOD via manually selected volumes of interest (VOIs) and expressed as standardized uptake values (SUV). Using SUVs for the tumor and brain regions the tumor to brain ratios were calculated.

## 5. Conclusions

Studied ^68^Ga-RGD peptides displayed excellent in vitro characteristics as well as in vivo behavior; on the contrary, ^68^Ga-DOTA-substance P failed in the in vivo stability test and was not able to image the tumor. In vivo comparison of ^68^Ga-RGD peptides with ^18^F-FDG and ^18^F-FLT in glioblastoma tumor model in mice revealed better imaging properties for ^68^Ga-RGD peptides. ^68^Ga-RGD peptides showed higher tumor to background ratio and a low accumulation in non-target organs except the excretory organs. These properties make ^68^Ga-RGD peptides, in particular, ^68^Ga-NODAGA-c(RGDyK)_2_, more suitable for preclinical imaging of GBM than clinically used ^18^F-tracers.

## Figures and Tables

**Table 1:** In vitro characterization of tested peptides. Partition coefficient (log P), human plasma protein binding and in vitro stability of ^68^Ga-peptides under various conditions presented as the mean of radiochemical purity ± standard deviation (n = 3 and n = 1 for ^68^Ga-DOTA-substance P) at different time points.

The Peptide	Log P	Incubation Time	Plasma Protein Binding (%)	% Stability in 0.1 M FeCl_3_	% Stability in 6 mM DTPA	% Stability in pH 7	% Stability in Plasma
^68^Ga-DOTA-c(RGDfK)	−3.43 ± 0.11	30 min	2.39	97.04 ± 1.24	99.39 ± 0.43	99.60 ± 0.23	99.23 ± 0.18
		60 min	2.65	96.76 ± 1.21	99.39 ± 0.54	99.51 ± 0.13	99.14 ± 0.19
		120 min	3.29	95.83 ± 1.04	99.06 ± 0.57	99.49 ± 0.20	98.81 ± 0.33
^68^Ga-NODAGA-c(RGDyK)	−3.05 ± 0.62	30 min	2.74	99.24 ± 0.44	99.65 ± 0.07	99.82 ± 0.05	99.23 ± 0.18
		60 min	1.31	98.56 ± 0.60	99.20 ± 0.37	99.78 ± 0.12	99.14 ± 0.19
		120 min	3.21	98.94 ± 0.42	99.23 ± 0.32	99.72 ± 0.22	98.81 ± 0.33
^68^Ga-NODAGA-c(RGDyK)_2_	−3.34 ± 0.13	30 min	1.74	98.66 ± 0.82	99.39 ± 0.43	99.83 ± 0.11	99.51 ± 0.17
		60 min	1.89	99.22 ± 0.19	99.39 ± 0.54	99.74 ± 0.18	99.31 ± 0.11
		120 min	3.40	99.17 ± 0.18	99.06 ± 0.57	99.62 ± 0.21	99.02 ± 0.33
^68^Ga-DOTA-substance P	−3.03 ± 0.33	30 min	22.35	88.4	73.4	98.2	98.2
		60 min	24.49	85.1	63.1	98.1	98.2
		120 min	29.21	78.8	56.1	98.1	98.0

**Table 2:** Tumor to blood, tumor to muscle and tumor to kidney ratios calculated from ex vivo biodistribution data for four studied ^68^Ga-peptides and two ^18^F-tracers. Every tracer was evaluated at two times points of 30 min and 90 min post-injection. Data are presented as mean values (n = 3).

	^68^GA-DOTA-C(RGDFK)		^68^GA-NODAGA-C(RGDYK)		^68^GA-NODAGA-C(RGDYK)2		^68^GA-DOTA-SUBSTANCE P		^18^F-FDG		^18^F-FLT	
	30 min p.i.	90 min p.i.	30 min p.i.	90 min p.i.	30 min p.i.	90 min p.i.	30 min p.i.	90 min p.i.	30 min p.i.	90 min p.i.	30 min p.i.	90 min p.i.
Tumor to blood ration	1.09	14.70	1.90	23.12	2.30	16.38	0.93	2.84	1.96	1.95	1.10	2.36
Tumor to muscle ration	2.42	13.53	3.23	6.51	5.84	10.47	3.10	6.45	2.67	3.58	1.31	2.83
Tumor to kidney ratio	0.29	1.43	0.36	0.90	0.61	0.91	0.14	0.04	1.01	2.21	0.76	1.79

**Table 3:** Tumor to brain ratios calculated from positron emission tomography (PET) data (standard uptake values (SUV) of respective regions) acquired 90 min p.i. of the tracers.

	^68^Ga-DOTA-c(RGDfK)	^68^Ga-NODAGA-c(RGDyK)	^68^Ga-NODAGA-c(RGDyK)_2_	^68^Ga-DOTA-Substance P	^18^F-FDG	^18^F-FLT
Tumor to brain ratio	4.70	4.00	4.14	4.25	0.64	4.58
